# Single-cell RNA-seq uncovers distinct pathways and genes in endothelial cells during atherosclerosis progression

**DOI:** 10.3389/fmolb.2023.1176267

**Published:** 2023-05-31

**Authors:** Min Wu, Yijin Wu, Shulin Tang, Jinsong Huang, Yueheng Wu

**Affiliations:** ^1^ Department of Cardiovascular Surgery, Guangdong Provincial People's Hospital (Guangdong Academy of Medical Sciences), Southern Medical University, China; ^2^ Medical Research Institute, Guangdong Provincial People's Hospital (Guangdong Academy of Medical Sciences), Southern Medical University, China

**Keywords:** atherosclerosis, single-cell RNA-seq, endothelial cells, fluid shear stress and atherosclerosis, TGF-beta

## Abstract

**Background:** Atherosclerosis (AS) is a chronic inflammatory disease involving various cell types, cytokines, and adhesion molecules. Herein, we aimed to uncover its key molecular mechanisms by single-cell RNA-seq (scRNA-seq) analysis.

**Methods:** ScRNA-seq data of cells from atherosclerotic human coronary arteries were analyzed using the Seurat package. Cell types were clustered, and differentially expressed genes (DEGs) were screened. GSVA (Gene Set Variation Analysis) scores of hub pathways were compared among different cell clusters. DEGs in endothelial cells between apolipoprotein-E (ApoE)^−/−^ mice and specific TGFbR1/2 KO ApoE^−/−^ mice fed with high-fat diet were overlapped with those from human AS coronary arteries. In fluid shear stress and AS, hub genes were determined based on the protein–protein interaction (PPI) network, which were verified in ApoE^−/−^ mice. Finally, hub genes were validated in three pairs of AS coronary arteries and normal tissues by histopathological examination.

**Results:** ScRNA-seq identified nine cell clusters in human coronary arteries, namely, fibroblasts, endothelial cells, macrophages, B cells, adipocytes, HSCs, NK cells, CD8^+^ T cells, and monocytes. Among them, endothelial cells had the lowest fluid shear stress and AS and TGF-beta signaling pathway scores. Compared to ApoE^−/−^ mice fed with normal diet, fluid shear stress and AS and TGF-beta scores were both significantly lower in endothelial cells from TGFbR1/2 KO ApoE^−/−^ mice fed with normal or high-fat diet. Furthermore, the two hub pathways had a positive correlation. Three hub genes (ICAM1, KLF2, and VCAM1) were identified, and their expression was distinctly downregulated in endothelial cells from TGFbR1/2 KO ApoE^−/−^ mice fed with normal or high-fat diet than in those from ApoE^−/−^ mice fed with a normal diet, which were confirmed in human AS coronary artery.

**Conclusion:** Our findings clarified the pivotal impacts of pathways (fluid shear stress and AS and TGF-beta) and genes (ICAM1, KLF2, and VCAM1) in endothelial cells on AS progression.

## Background

Atherosclerosis (AS) is a chronic arterial disease, which is the main cause of cardiovascular disease and stroke (Solanki et al., 2018). AS often occurs in medium- and large-sized arteries composed of endothelial cells, vascular smooth muscle cells, and other vascular cells. Among them, endothelial cells are the main cell type, which are constantly exposed to shear stress from the bloodstream (Niu et al., 2019). Thus, shear stress regulates the structure and function of the endothelium. Mechanically sensitive transcription factors [such as kruppel-like factor 2 (KLF2)] are considered promising therapeutic targets for prevention and treatment of AS (Niu et al., 2019). Endothelial dysfunction is an early indicator of AS, characterized by overexpression of adhesion molecules, including intercellular adhesion molecule-1 (ICAM-1) and vascular cell adhesion molecule-1 (VCAM-1) (Habas and Shang, 2018). Endothelial-to-mesenchymal transition (EndMT) can cause endothelial dysfunction, which is prominent in AS (Hao et al., 2019). Targeting EndMT may be a promising treatment strategy for AS (Kovacic et al., 2019). The transforming growth factor-β (TGF-β) signaling pathway is an effective inducer of EndMT (Sabbineni et al., 2018). Dysfunctional endothelial cells facilitate the adhesion and migration of leukocytes into the blood vessel wall. Once under the endothelium, monocytes differentiate into macrophages. Activation of endothelial cells and infiltration of circulating monocytes into the vessel wall contribute to the progression of AS (Zhuang et al., 2019).

Several novel treatments are currently being developed. A healthy lifestyle can reduce the risk of AS. Statins are considered the main drug treatment strategy, which can lower plasma low-density lipoprotein (LDL) levels. Although statins have been shown to be effective, individual responses to these drugs vary greatly (Ray et al., 2017). However, there are still challenges in regaining normal physiological conditions (Hurtubise et al., 2016). Therefore, it is necessary to develop other therapies. Recent clinical and preclinical studies have demonstrated that targeted therapy of AS-related targets is a promising treatment. For example, oligonucleotides targeting human ANGPTL3 can delay the progression of AS (Graham et al., 2017). Furthermore, inclisiran (a siRNA targeting PCSK9 mRNA) can reduce LDL cholesterol levels in patients with high cardiovascular risk (Ray et al., 2017). Therefore, in-depth exploration of AS-related genes and pathways will help promote the application of RNA-based treatments in AS.

The traditional transcriptome uses bulk sequencing, processes thousands of cells at the same time, and obtains their average value. However, no two cells have the same level of gene expression. Emerging single-cell transcriptome sequencing technology can reveal the differences between individual cells and find the heterogeneity among individual cells. In addition, this technology can identify rare and easily overlooked cell populations and more deeply clarify the molecular mechanism of disease. ScRNA-seq has been widely used in the field of cardiovascular disease research (Yifan et al., 2020). ScRNA-seq provides a detailed transcriptional picture of different cell types in AS (Deng et al., 2020). Herein, we analyzed scRNA-seq of cells from atherosclerotic human coronary arteries and identified different cell subpopulations for AS. Among them, endothelial cells had the lowest GSVA scores of two hub pathways (fluid shear stress and AS and TGF-beta signaling pathway). These hub pathways were further validated in endothelial cells between ApoE^−/−^ mice and specific TGFbR1/2 KO ApoE^−/−^ mice fed with high-fat diet. Furthermore, three hub genes (ICAM1, KLF2, and VCAM1) were identified, which were verified in three pairs of AS coronary arteries and normal tissues by immunofluorescence. Therefore, our findings provide novel insights into the molecular mechanisms of AS via scRNA-seq.

## Materials and methods

### scRNA-seq acquisition

ScRNA-seq data from the atherosclerotic area of the proximal to mid right coronary arteries of four heart transplant recipients were retrieved from the Gene Expression Omnibus (GEO; https://www.ncbi.nlm.nih.gov/gds/) database (accession number: GSE131778) (Wirka et al., 2019) on the Illumina HiSeq 4000 GPL20301 platform. RNA-seq profiles of endothelial cells isolated from the whole aorta between ApoE^−/−^ mice and specific TGFbR1/2 KO ApoE^−/−^ mice fed with high-cholesterol high-fat diet (HCHFD; 40% kcal% fat, 1.25% cholesterol, and 0% cholic acid) for 16 weeks were obtained from the GSE134557 dataset (Chen et al., 2019). The significant markers of mice were converted to human genes through the Ensembl BioMart tool (http://asia.ensembl.org/index.html) and overlapped with the marker genes in the GSE131778 dataset.

### Quality control and data filtering

10x Genomics was imported into the Seurat package in R (Butler et al., 2018). The dataset was trimmed of cells expressing <100 genes and genes expressed in <5 cells. Low-quality cells, such as those in a necrotic state and undergoing degradation of RNA in the cell, were filtered. Each gene was normalized by the unique molecular identifiers (UMI) < 100 of the gene/the total UMI of the intracellular transcript ×10,000. Through the FindVariableGenes algorithm, the average expression of each gene and its dispersion were calculated. Since not all genes had valid information, highly variable genes were calculated with the filtering conditions of 0.0125< average expression >3 and dispersion >0.5.

### Principal component analysis (PCA)

Linear dimensionality reduction was presented utilizing the RunPCA function in the Seurat package (Andrews and Hemberg, 2018). The optimal number of principal components (PCs) was identified utilizing the PCElbowPlot function.

### Differential expression analysis and cell cluster

The PCs with cumulative standard deviation >70% were selected for cell clustering analysis by the FindNeighbors and FindClusters functions in the Seurat package. Then RunUMAP function was utilized for uniform manifold approximation and projection (UMAP) (Becht et al., 2018). Using the FindAllMarkers function, differentially expressed genes (DEGs) were obtained according to log |fold change (FC)| ≥ 0.25, the expression ratio of cell population ≤0.25, and *p*-value ≤0.05. According to DEGs, cell subtypes were identified through the CellMarker database (http://biocc.hrbmu.edu.cn/CellMarker/) (Zhang et al., 2019) and the findmarkergenes function of single-cell Cluster-based Annotation Toolkit for Cellular Heterogeneity (scCATCH, https://github.com/ZJUFanLab/scCATCH) (Shao et al., 2020).

### Functional enrichment analysis

Gene ontology (GO) analysis was presented via the clusterProfiler package in R (Yu et al., 2012). Kyoto Encyclopedia of Genes and Genomes (KEGG) enrichment analysis was performed by the Metascape online database (Zhou et al., 2019) in line with min Overlap = 3, *p*-value cutoff = 0.05, and min enrichment = 1.5.

### Gene set variation analysis (GSVA)

GSVA is a non-parametric unsupervised analysis method that is used to evaluate the results of gene set enrichment of microarray or RNA-seq (Hänzelmann et al., 2013). By integrating KEGG (http://www.genome.ad.jp/kegg/) (Ogata et al., 1999), REACTOME (https://reactome.org/) (Fabregat et al., 2018), and PathCards databases (http://pathcards.genecards.org/) (Belinky et al., 2015), the gene list of hub pathways was collected. The expression matrix of genes among different cell clusters was transformed into the expression matrix of gene sets among cell clusters. Enriched pathways were scored by the GSVA algorithm.

### Protein–protein interaction (PPI) network

Genes enriched in the fluid shear stress and AS pathway were extracted for construction of a PPI network through the STRING database (version 11; https://string-db.org/), with a cutoff value of 0.4 (Szklarczyk et al., 2019). Cytoscape software (version 3.7.2) was utilized to calculate the degree of each node (Doncheva et al., 2019). The correlation coefficient between the GSVA score of hub pathways and the expression value of the enriched genes in the sample was calculated through Spearman’s correlation analysis. The correlation coefficient > 0.4 and *p*-value < 0.05 were considered to be statistically related. Hub genes with high degree and strong correlation with the GSVA score of hub pathways were selected.

### Patients and specimens

A total of three pairs of AS left coronary arteries and normal tissues were harvested from patients with severe atherosclerotic coronary artery diseases who received heart transplantations at Guangdong General Hospital according to an Institutional Review Board-–approved protocol (Institutional Review Board No. GDREC2016255H). This study strictly followed the ethical principles of medical research involving human subjects according to the Declaration of Helsinki (2013 version). Our study was approved by the Ethics Committee of the Guangdong Provincial People’s Hospital. All clinical research subjects signed written informed consent.

### Western blot

Coronary artery tissues were lysed with RIPA lysis buffer (Millipore, United States) plus protease and phosphatase inhibitors. Protein concentration was evaluated using a BCA protein assay kit (Beyotime, Shanghai, China). Extracted protein was separated via 10% SDS-PAGE and transferred onto a polyvinylidene difluoride membrane (Millipore, United States). Afterward, the membrane was sealed by 5% milk/TBST and incubated with primary antibodies against VCAM1 (1:1,000; ab115135; Abcam, United States), KLF2 (1:1,000; ab236507; Abcam, United States), ICAM1 (1:1,000; ab282575; Abcam, United States), and GAPDH (1:1000; ab8245; Abcam, United States) at 4°C overnight. Then, the membrane was incubated with horseradish peroxidase (HRP)-conjugated secondary antibody (1:2000; ab7090; Abcam, United States) at room temperature for 2 h. The bands were developed through an ECL kit (Bio-Rad, United States). The expression of target proteins was quantified with ImageJ software.

### Real-time quantitative polymerase-chain reaction (RT-qPCR)

RNA was extracted from coronary artery tissues through TRIzol (Invitrogen, United States). Total RNA was then reverse-transcribed to cDNA according to the following procedure: at 25°C for 5 min, at 42°C for 30 min, and at 85°C for 5 min. RT-qPCR was performed on the RT-qPCR instrument (ABI QuantStudio™ 12K Flex; ABI, United States). Primer sequences were as follows: GAPDH: 5′-CTG​GGC​TAC​ACT​GAG​CAC​C-3′ (forward) and 5′-AAG​TGG​TCG​TTG​AGG​GCA​ATG-3′ (reverse); VCAM1: 5′-GGG​AAG​ATG​GTC​GTG​ATC​CTT-3′ (forward) and 5′-TCT​GGG​GTG​GTC​TCG​ATT​TTA-3′ (reverse); KLF2: 5′-TTC​GGT​CTC​TTC​GAC​GAC​G-3′ (forward) and 5′-TGC​GAA​CTC​TTG​GTG​TAG​GTC-3′ (reverse); ICAM1: 5′-ATG​CCC​AGA​CAT​CTG​TGT​CC-3′ (forward) and 5′-GGG​GTC​TCT​ATG​CCC​AAC​AA-3′ (reverse). GAPDH served as an internal control. Relative mRNA expression was calculated by the 2^−ΔΔCt^ method.

### Hematoxylin–eosin (H&E)

The coronary artery tissues were fixed overnight in paraformaldehyde solution (Seville, Wuhan, China). After dehydration, the specimens were embedded in paraffin. The paraffin sections were cut to a thickness of 5 μm. The paraffin sections were deparaffinized with xylene and a series of concentrations of ethanol solutions. Then, the sections were stained with Harris hematoxylin solution (Beyotime, Beijing, China) for 5 min. After differentiation by 1% hydrochloric acid alcohol, the sections were stained with eosin staining solution for 2 min. The sections were treated with 70%, 80%, and 95% ethanol and anhydrous ethanol for dehydration. After placing it in xylene I for 2 min and xylene II for 2 min, neutral gum was used to mount the film. Images were observed under a microscope (Olympus, Japan).

### EVG staining

The deparaffinized sections were stained with Verhoeff’s staining solution for 1–3 min at room temperature. After differentiation with 2% ferric chloride solution for 10–20 s, Van Gieson’s solution was used to counter-stain for 10–15 s. Following mounting the slides by neutral gum, images were observed under a microscope.

### Masson staining

After the paraffin sections were dewaxed with xylene and a series of concentrations of ethanol, they were immersed in potassium dichromate solution overnight. The sections were placed in iron hematoxylin staining solution for 3 min. Following differentiation via 1% hydrochloric acid alcohol, the sections were immersed in Ponceau acid fuchsin staining solution for 5 min. Then, the sections were stained with aqueous phosphomolybdic acid solution for 3 min and aniline blue staining solution for 5 min. After washing with 1% glacial acetic acid for 1 min, the sections were dehydrated by absolute ethanol and then clarified by xylene for 5 min. Neutral gum was used to mount the slides, and images were observed under a microscope.

### Immunofluorescence

The deparaffinized sections were incubated with Tris–EDTA buffer for 20 min and 3% H_2_O_2_ for 10 min. The sections were exposed to 2% BSA for 30 min at 37°C. Then, they were incubated with fluorescein-labeled primary antibodies against ICAM1 (1:200; ab109361; Abcam, United States), KLF2 (1:200; ab244507; Abcam, United States), and VCAM1 (1:200; ab134047; Abcam, United States) for 4 h at room temperature. The results were investigated under an immunofluorescence microscope (Olympus, Japan).

### Statistical analysis

The experiment results are expressed as mean ± standard deviation. Comparisons between two groups were presented with Student’s *t*-test. *p*-value < 0.05 indicated statistical significance.

## Results

### Dimensionality reduction analysis of scRNA-seq data from human AS coronary artery

**FIGURE 1:**
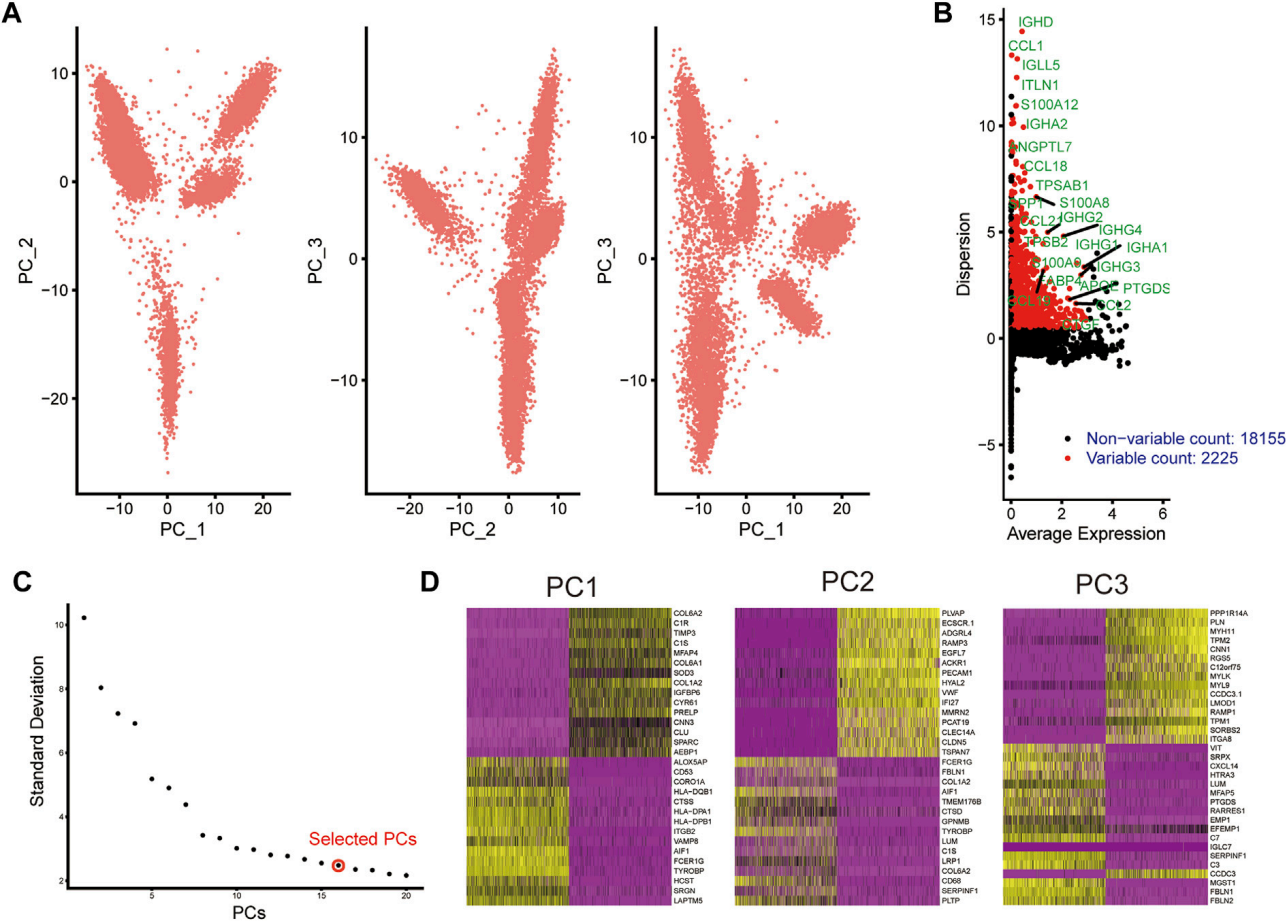
Dimensionality reduction analysis of scRNA-seq data from human AS coronary artery. **(A)** PCA plots of scRNA-seq data. Each dot represents a cell. **(B)** Marking the 25 most highly variable genes after normalization. The abscissa expresses the average expression of each gene, and the ordinate expresses the Z-value of dispersion. **(C)** Determination of the optimal number of PCs. **(D)** Heat maps depicting the top 30 genes in each PC.

scRNA-seq data from human AS coronary artery were retrieved from the GSE131778 dataset. Following quality control and filtration, scRNA-seq data were linearly scaled (Figure 1A). Totally 2225 highly variable genes were identified having a high degree of differences based on their average expression and dispersion, due to biological effects rather than just technical noise (Figure 1B). The inflection point of standard deviation appeared when PC = 16 (Figure 1C). Thus, we chose 15 genes for cluster analysis. Figure 1D shows the top 30 genes in each PC.

### Identification of cell subtypes and biological functions of DEGs for AS

**FIGURE 2:**
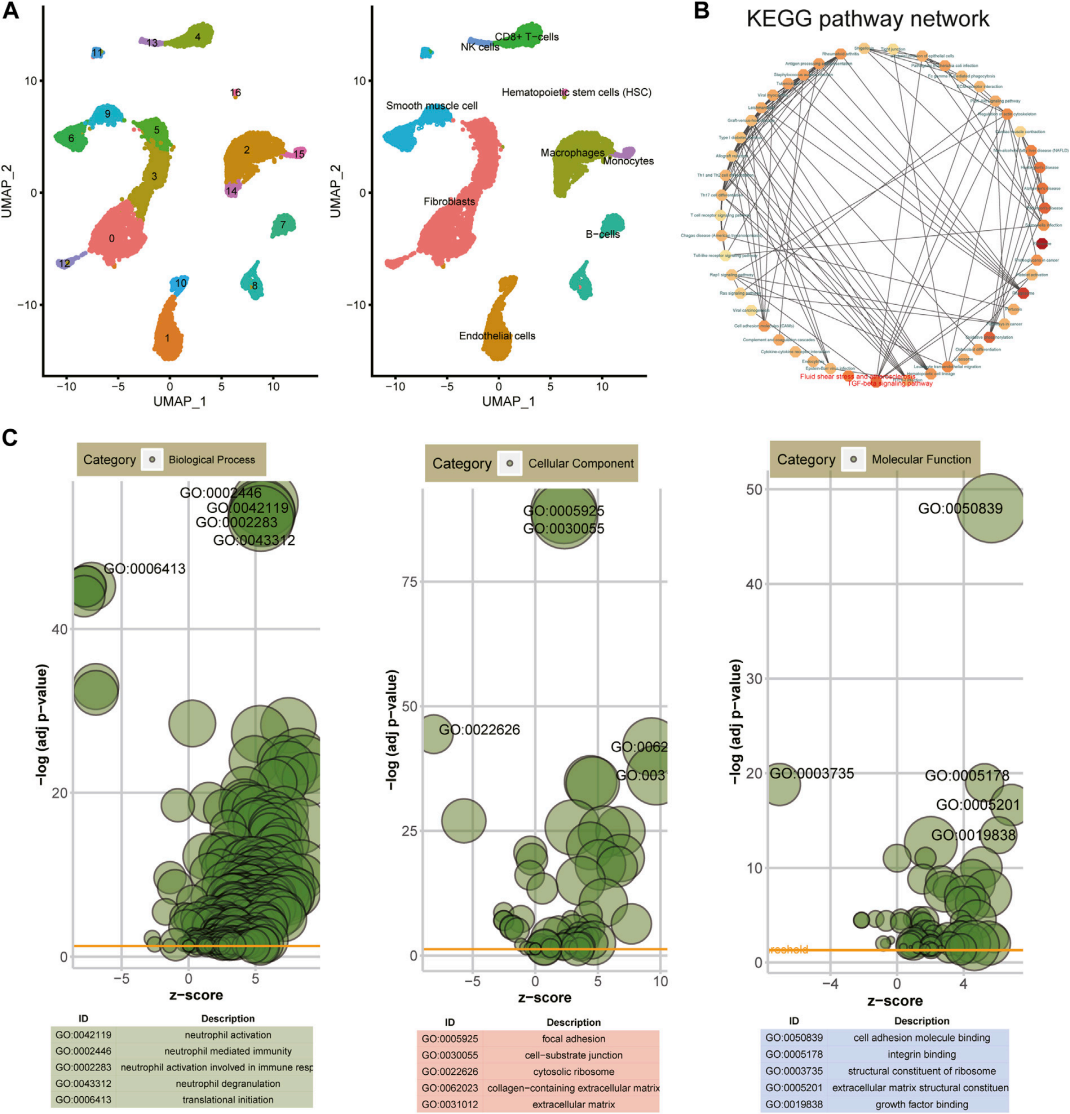
Identification of cell subtypes and biological functions of DEGs. **(A)** UMAP clustering of cells from atherosclerotic human coronary arteries. **(B)** KEGG pathway network of DEGs via the Metascape database. **(C)** Bubble charts depicting the top five enrichment results for GO—biological process, cellular component, and molecular function.

Based on the UMAP clustering method, all cells were clustered into nine cell subtypes, namely, fibroblasts, endothelial cells, macrophages, B cells, adipocytes, hematopoietic stem cells (HSC), NK cells, CD8^+^ T cells, and monocytes (Figure 2A). KEGG and GO enrichment analysis of DEGs were then carried out. In the KEGG pathway network, fluid shear stress and AS and TGF-beta signaling pathways were the most significantly enriched by these DEGs, which were considered the two hub pathways (Figure 2B). Furthermore, immune-related pathways were also distinctly enriched, like Th1 and Th2 cell differentiation, Th17 cell differentiation, complement and coagulation cascades, and cytokine–cytokine receptor interaction. For GO-biological processes, these DEGs were distinctly enriched in immune responses like neutrophil activation, neutrophil-mediated immunity, neutrophil activation involved in immune response, neutrophil degranulation, and translational initiation (Figure 2C). These genes were involved in key cellular components of AS such as focal adhesion, cell–substrate junction, cytosolic ribosome, collagen-containing extracellular matrix, and extracellular matrix. In addition, they might possess crucial molecular functions, including cell adhesion molecular binding, integrin binding, structural constituent of ribosome, and extracellular matrix structural constituent.

### AS endothelial cells have the lowest GSVA scores of hub pathways among different cell clusters

**FIGURE 3:**
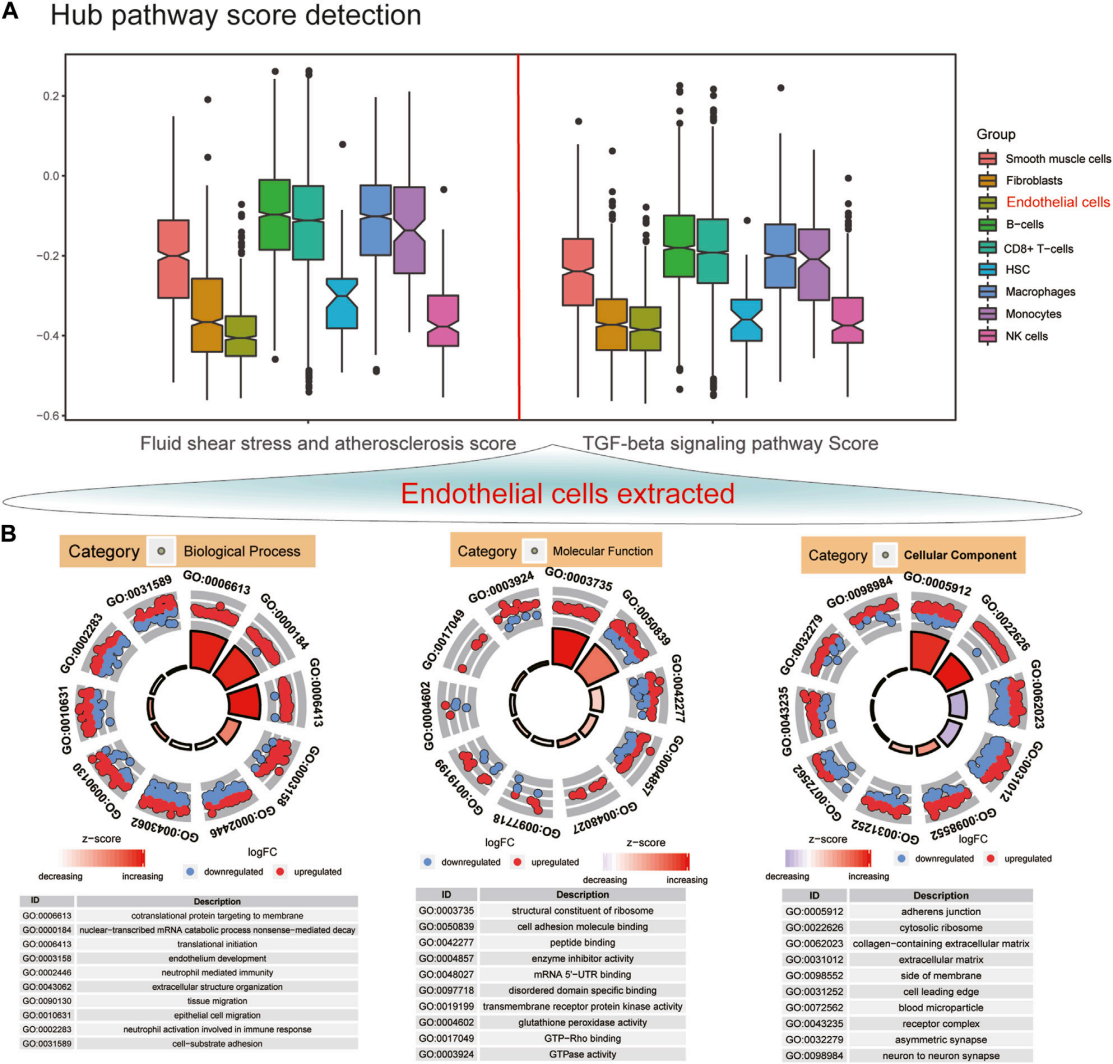
Endothelial cells have the lowest GSVA scores of hub pathways among different cell clusters. **(A)** GSVA score detection of fluid shear stress and atherosclerosis and TGF-beta signaling pathway in AS endothelial cells in different cell clusters. **(B)** Top 10 GO enrichment analysis results of DEGs in endothelial cells including biological process, molecular function, and cellular component. Red dots represent upregulated genes, and blue dots represent downregulated genes.

GSVA was utilized to score the two hub pathways in different cell clusters. Figure 3A shows the lowest GSVA scores of fluid shear stress and AS and TGF-beta signaling pathway in endothelial cells among nine cell clusters. Then, we further analyzed the biological functions of DEGs in endothelial cells. These genes were significantly involved in pivotal biological processes like cotranslational protein targeting to membrane, nonsense-mediated decay of nuclear-transcribed mRNA catabolic process, translational initiation, and endothelium development (Figure 3B). Moreover, they might mediate important molecular functions of the structural constituent of ribosome, cell adhesion molecular binding, peptide binding, enzyme inhibitor activity, and mRNA 5′-UTR binding. Furthermore, these DEGs could participate in regulation of various cellular components, especially for adherens junction and the cytosolic ribosome.

### Identification of common marker genes in endothelial cells between human AS and ApoE^−/−^ mice model

**FIGURE 4:**
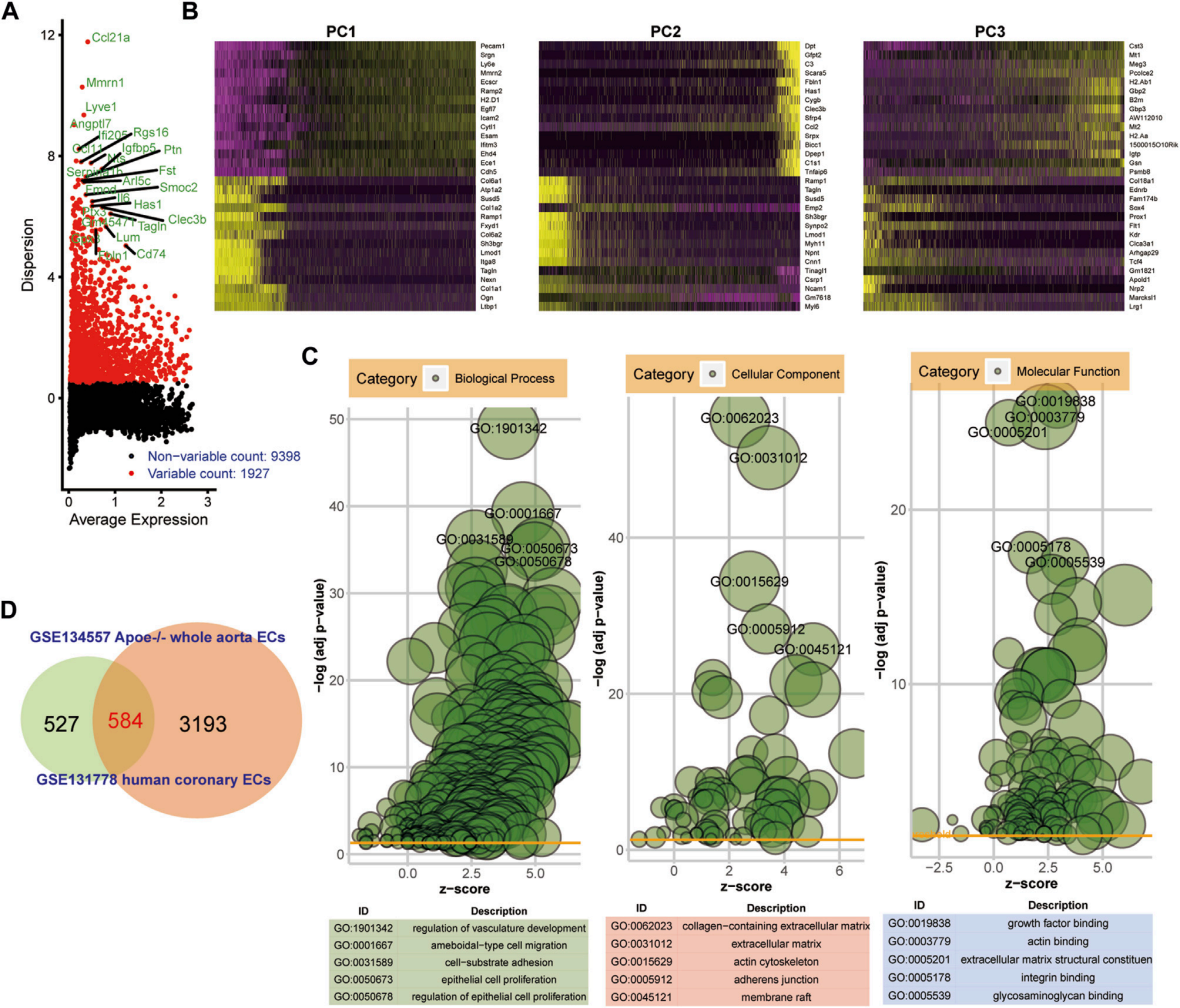
Identification of common marker genes in endothelial cells between human AS and ApoE^−/−^ mice model. **(A)** Highly variable genes in ApoE^−/−^ mice endothelial cells. **(B)** Heat maps depicting the top 30 genes in the first three PCs. **(C)** Bubble diagrams showing the most significantly enriched 10 GO enrichment analysis results including biological process, cellular component, and molecular function. **(D)** Venn diagram depicting 584 common genes between human AS coronary endothelial cells and ApoE^−/−^ mice endothelial cells.

ScRNA-seq data from endothelial cells between ApoE^−/−^ mice and TGFbR1/2 KO ApoE^−/−^ mice fed with high-fat diet were obtained from the GSE134557 dataset. After quality control and data filtration, scRNA-seq data were linearly scaled by PCA. In total, 1927 highly variable genes were identified (Figure 4A). Figure 4B exhibited the top 30 genes in the top three PCs. Marker genes were significantly enriched in AS-related biological processes, including regulation of vascular development, ameboid-type cell migration, cell-substrate adhesion, epithelial cell proliferation, and regulation of epithelial cell proliferation (Figure 4C). These marker genes were distinctly related to several key cellular components like collagen-containing extracellular matrix, extracellular matrix, actin cytoskeleton, adherens junction, and membrane raft. In addition, they could be involved in regulating crucial molecular functions of growth factor binding, actin binding, extracellular matrix structural constituent, integrin binding, and glycosaminoglycan binding. Genes in human AS coronary endothelial cells and ApoE^−/−^ mice whole aorta endothelial cells were overlapped. As a result, 584 common genes were obtained (Figure 4D).

### Hub pathways and genes in endothelial cells for AS

**FIGURE 5:**
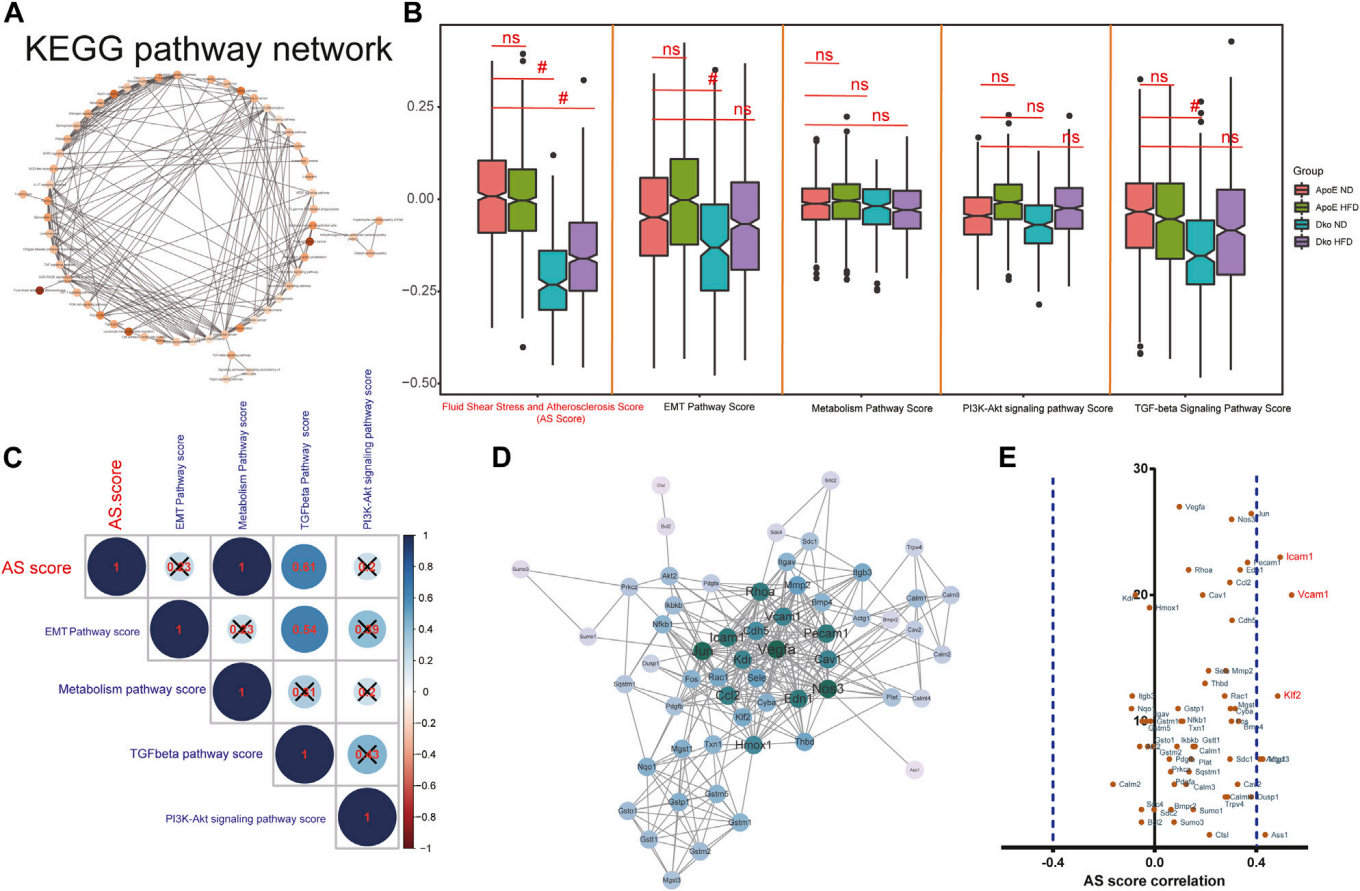
Hub pathways and genes in endothelial cells for AS. **(A)** KEGG pathway network of 584 common genes. **(B)** GSVA scores of fluid shear stress and atherosclerosis, EMT pathway, metabolism pathway, PI3K-Akt signaling pathway, and TGF-beta signaling pathway in endothelial cells from four groups (ApoE^−/−^ mice fed with normal diet or high-fat diet, Dko (double knock out of TGFbR1/2 and ApoE) mice fed with normal diet or high-fat diet). Ns, no statistical significance; #*p* < 0.05. **(C)** Correlation analysis results between different hub pathways. **(D)** PPI network of genes in fluid shear stress and atherosclerosis. **(E)** Pearson correlation analysis results between marker gene expression and fluid shear stress and atherosclerosis score.

**FIGURE 6:**
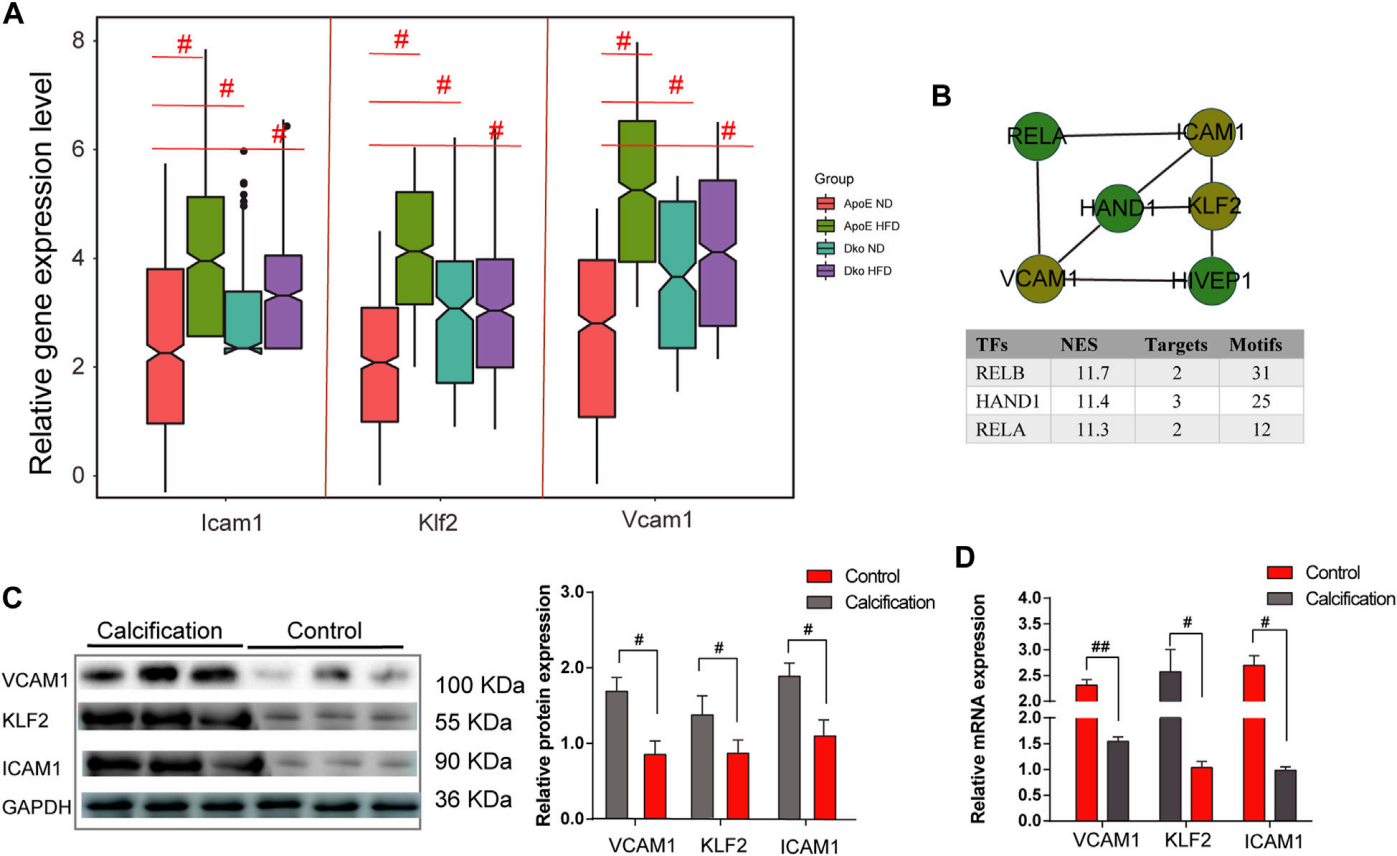
Identification and validation of hub genes in AS. **(A)** Relative expression levels of ICAM1, KLF2, and VCAM1 in endothelial cells from four groups. **(B)** Prediction of transcription factors for ICAM1, KLF2, and VCAM1. **(C)** Western blot validating the protein expression of ICAM1, KLF2, and VCAM1 in human AS coronary artery and normal tissues. **(D)** RT-qPCR validating the mRNA expression of ICAM1, KLF2, and VCAM1 in human AS coronary artery and normal tissues. #*p* < 0.05.

Altogether, 584 common genes were utilized for KEGG enrichment analysis. As shown in Figure 5A, fluid shear stress and AS, EMT pathway, metabolism pathway, PI3K–Akt signaling pathway, and TGF-beta signaling pathway were the most significantly enriched, which were considered hub pathways. Furthermore, we also found that these genes were distinctly enriched in several immune responses like the chemokine signaling pathway, IL-17 signaling pathway, and Th17 cell differentiation. We then calculated the GSVA scores of hub pathways in different groups. Compared to ApoE^−/−^ mice fed with a normal diet, the GSVA score of fluid shear stress and AS was distinctly lower in endothelial cells from TGFbR1/2 KO ApoE^−/−^ mice fed with normal or high-fat diet (*p* < 0.05; Figure 5B). However, no significant difference in its score was found between ApoE^−/−^ mice fed with normal diet and those fed with high-fat diet. The EMT pathway score was distinctly decreased in endothelial cells from TGFbR1/2 KO ApoE^−/−^ mice fed with normal diet in comparison to those from ApoE^−/−^ mice fed with normal diet (*p* < 0.05). Compared to endothelial cells from ApoE^−/−^ mice fed with normal diet, its score was not statistically differential in endothelial cells from ApoE^−/−^ mice or TGFbR1/2 KO ApoE^−/−^ mice fed with high-fat diet. In comparison to ApoE^−/−^ mice fed with normal diet, there was no significant difference in metabolism pathway and PI3K–Akt signaling pathway scores in ApoE^−/−^ mice fed with high-fat diet or TGFbR1/2 KO ApoE^−/−^ mice fed with normal or high-fat diet. There was a significantly lower TGF-beta signaling pathway score in endothelial cells from TGFbR1/2 KO ApoE^−/−^ mice fed with normal diet than in those from ApoE^−/−^ mice fed with normal diet. No statistical difference was detected between ApoE^−/−^ mice fed with normal diet and ApoE^−/−^ mice or TGFbR1/2 KO ApoE^−/−^ mice fed with high-fat diet. In Figure 5C, the Pearson correlation analysis results showed that fluid shear stress and AS and EMT pathway scores were both positively correlated to the TGF-beta signaling pathway score (*r* = 0.61 and *p* < 0.05; *r* = 0.54 and *p* < 0.05, respectively). Genes in the fluid shear stress and AS were extracted for PPI network construction through the STRING database. Using the Cytoscape software, we calculated the degree of each node. As shown in Figure 5D, Icam1, Klf2, Vcam1, Ccl2, Pecam1, Cav1, Vegfa, Phoa, Jun, and Hmox1 had the highest degree in the PPI network. The Spearman correlation analysis results showed that Icam1, Klf2, and Vcam1 were significantly positively correlated to a higher degree of PPI network and AS score (Figure 5E). We further analyzed the difference in Icam1, Klf2, and Vcam1 expression in different groups. As shown in Figure 6A, Icam1, Klf2, and Vcam1 expression was significantly downregulated in endothelial cells from TGFbR1/2 KO ApoE^−/−^ mice fed with normal or high-fat diet than those from ApoE^−/−^ mice fed with normal diet (*p* < 0.05). No significant difference in their expression was found in endothelial cells between ApoE^−/−^ mice with normal diet and those with high-fat diet. Thus, Icam1, Klf2, and Vcam1 were selected as hub genes. Their transcription factors were predicted via the plug-in iRegulon for Cytoscape software. As shown in Figure 6B, RELB (NES = 11.7, targets = 2, and motifs = 31), HAND1 (NES = 11.4, targets = 3, and motifs = 25), and RELA (NES = 11.3, targets = 2, and motifs = 12) were potential transcription factors for Icam1, Klf2, and Vcam1, respectively.

### Validation of hub genes in human AS coronary artery tissues

**FIGURE 7:**
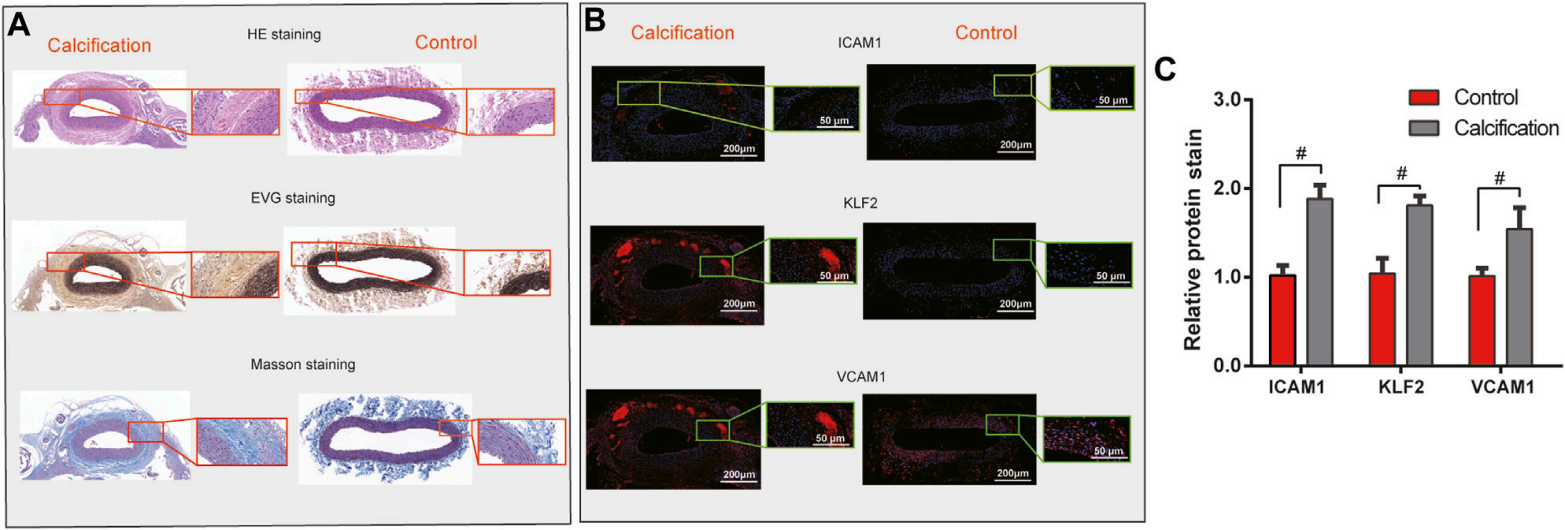
Validation of hub genes in human AS coronary artery tissues. **(A)** H & E, EVG, and Masson staining results of human AS coronary artery and normal tissues. For H & E staining, the nucleus was stained blue, and the cytoplasm was stained red. For EVG staining, elastic fibers and nuclei were dyed black, and the collagen fibers were dyed red. For Masson staining, collagen fibers and cell nuclei were stained blue, and the muscle fibers and cellulose were stained red. **(B,C)** Immunofluorescence detecting ICAM1, KLF2, and VCAM1 expression in human AS coronary artery and normal tissues. Scale bar = 200 μm. Magnification: ×4. #*p* < 0.05.

Three pairs of human AS coronary arteries and normal tissues were collected in this study. Western blot was used to validate the protein expression of hub genes including ICAM1, KLF2, and VCAM1. As a result, we observed that ICAM1, KLF2, and VCAM1 (all *p* < 0.05) were all highly expressed in the AS coronary artery than in normal tissues (Figure 6C). Moreover, our RT-qPCR results showed significant downregulation of ICAM1 and VCAM1 mRNA (both *p* < 0.05) as well as significant upregulation of KLF2 mRNA (*p* < 0.05) in the AS coronary artery compared to normal tissues (Figure 6D). Coronary H&E staining results showed that there was no atheromatous plaque deposition on the coronary vessel wall in the normal controls (Figure 7A). Atherosclerotic plaque deposits were observed on the coronary vessel wall of the AS group. Foam cells were found on the surface of the plaque, the fiber cap was thin and uneven, and the surface of the plaque was eroded and peeled off. The membrane was thinner. The number of smooth muscle cells was reduced, and calcified granules were scattered. There was adventitial capillary angiogenesis, fibrous connective tissue hyperplasia, and plasma cell and lymphocyte infiltration. Coronary artery EVG staining results demonstrated that compared with those in the normal control group, in the AS coronary artery tissues, the thickened inner membrane contained large elastic fibers and collagen fibers. The inner elastic plate was broken, the ratio of the media was reduced, and the elastic fibers of the media were broken (Figure 7A). As shown in the Masson staining results, in comparison to the normal control group, there was lumen stenosis, intimal proliferation, increased intimal cells, disordered arrangement, and irregular inner elastic membrane in the coronary artery in the AS group (Figure 7A). Moreover, there were smooth muscle cells and collagen fibers in the thickened intima. Our immunofluorescence results confirmed that ICAM1, KLF2, and VCAM1 expression (all *p* < 0.05) was distinctly downregulated in AS coronary artery tissues compared to controls (Figures 7B, C).

## Discussion

ScRNA-seq has been widely applied in cardiovascular disease research, including AS (Chaudhry et al., 2019). ScRNA-seq can be used to clarify cell subsets and transcriptional changes under normal physiological and pathophysiological conditions. ScRNA-seq allows the separation of various cell subpopulations and the identification of phenotypic heterogeneity within a population of single-cell subtypes. Recently, immune cell heterogeneity has been evaluated in the arterial wall of AS mice by scRNA-seq (Winkels et al., 2018). The pathological mechanism of AS is huge and complex and involves a variety of cells and molecules. In specific cell types, changes in RNA expression levels and abnormal signaling pathways have been identified as key factors in the pathological basis of AS. Herein, we identified key pathways (fluid shear stress and AS and TGF-beta) and hub genes (ICAM1, KLF2, and VCAM1) in endothelial cells during AS progression by scRNA-seq analysis, which deepened our understanding on the molecular mechanism of AS.

In this study, scRNA-seq identified nine cell clusters in the coronary artery of AS patients, namely, fibroblasts, endothelial cells, macrophages, B cells, adipocytes, hematopoietic stem cells (HSC), NK cells, CD8^+^ T cells, and monocytes. Among them, endothelial cells had the lowest fluid shear stress and AS and TGF-beta signaling pathway scores. Compared to ApoE^−/−^ mice fed with normal diet, fluid shear stress and AS score was significantly lower in endothelial cells from TGFbR1/2 KO ApoE^−/−^ mice fed with normal diet or high-fat diet. In addition, EMT and TGF-beta signaling pathway scores were both suppressed in endothelial cells from TGFbR1/2 KO ApoE^−/−^ mice fed with normal diet. We found that fluid shear stress and AS score and EMT pathway score were both positively correlated to the TGF-beta signaling pathway score. Endothelial cells can sense hemodynamic changes imposed by blood flow regulation (Baratchi et al., 2017). Endothelial cells have no cilia in areas of high shear stress, but have cilia in areas of oscillating flow (Zaragoza et al., 2012). The high shear stress and non-ciliated endothelial cells are consistent with the EndMT region. These endothelial cells release growth factors, including TGF-β (Goumans and Ten Dijke, 2018). The changes in mechanical force at the blood–endothelial interface caused by shearing may also lead to changes in the morphology of endothelial cell monolayers and rearrangement of the endothelial cytoskeleton during AS. These changes depend on the activation of TGF-β, KLF2, and EndMT (Chen et al., 2019). AS is caused by damage to the blood vessel wall, and TGF-β is most likely to regulate the fibrotic and inflammatory components of the disease. TGF-β is produced by vascular cells and inflammatory cells. TGF-β stimulates EC proliferation, migration, and tube formation (Chen et al., 2019). Endothelial cells will experience EndMT when stimulated by TGF-β (Goumans and Ten Dijke, 2018). Thus, therapeutic interventions that interfere with TGF-β signaling are an emerging area under investigation.

Adhesion molecules mediate cell-to-cell and cell-to-extracellular matrix contact and adhesion, which participate in cell activation, signal transduction, proliferation, differentiation, and inflammation (Zhu et al., 2018). Moreover, they could be involved in the formation of AS plaques. The immunoglobulin superfamily (IgSF) is one of the adhesion molecules associated with AS. ICAM-1 and VCAM-1 are key family members of IgSF. Their high expression induces proliferative capacity of macrophages. The accumulation of abundant macrophages further accelerates the instability of AS plaques. In AS plaques, high expression of VCAM-1 and ICAM-1 facilitates the formation of new blood vessels. In addition, VCAM-1 may be expressed in the later stages of AS. Endothelial KLF2 can regulate the expression of various anti-inflammatory, anti-oxidant, and anti-thrombosis genes in endothelial cells (Sweet et al., 2018). KLF2 can be maintained by laminar shear stress (LSS) and induced by simvastatin. Oscillatory shear stress (OSS) reduces the expression of KLF2 in the endothelium, thereby promoting the activation of inflammasomes in the endothelium and the formation of AS. LSS or simvastatin reduces vascular inflammation by increasing the expression of KLF2 in the endothelium, thereby slowing down the inhibitory effect on AS. Further *in vivo* studies have shown that KLF2 protects aortic AS (Zhou et al., 2012). Consistently, our results demonstrated that VCAM-1, ICAM-1, and KLF2 had the highest correlation with fluid shear stress and AS score. Their expression was distinctly inhibited in endothelial cells from TGFbR1/2 KO ApoE^−/−^ mice fed with normal diet or high-fat diet than in cells from ApoE^−/−^ mice fed with normal diet. Under prediction, RELB, HAND1, and RELA could mediate the transcription of Icam1, Klf2, and Vcam1. According to previous reports, RELB, HAND1, and RELA are critical transcriptional factors in cardiovascular disease, and RELB (Mao et al., 2019) and RELA (Xie et al., 2018) have been confirmed to be related to AS progression. The underlying mechanisms of how these factors regulate the expression of Icam1, Klf2, and Vcam1 should be further validated through *in vitro* experiments.

Taken together, we uncovered distinct pathways and genes in endothelial cells during AS progression by scRNA-seq analysis. Although the expression of hub genes was validated in human AS coronary artery, their biological roles should be observed in a larger population in future studies.

## Conclusion

In this study, we revealed crucial roles of hub pathways (fluid shear stress and AS and TGF-beta) and genes (ICAM1, KLF2, and VCAM1) in endothelial cells on AS progression, which deserve more in-depth exploration.

## Data Availability

The original contributions presented in the study are included in the article/Supplementary Material; further inquiries can be directed to the corresponding author.

## Abbreviations

ApoE, apolipoprotein-E; AS, atherosclerosis; DEGs, differentially expressed genes; EndMT, endothelial-to-mesenchymal transition; FC, fold change; GEO, Gene Expression Omnibus; GO, Gene Ontology; GSVA, Gene Set Variation Analysis; H & E, hematoxylin–eosin; ICAM-1, intercellular adhesion molecule-1; KEGG, Kyoto Encyclopedia of Genes and Genomes; KLF2, kruppel-like factor 2; LDL, low-density lipoprotein; PCA, principal component analysis; PCs, principal components; PPI, protein–protein interaction; scRNA-seq, single-cell RNA-seq; TGF-β, transforming growth factor-β; VCAM-1, vascular cell adhesion molecule-1; UMI, unique molecular identifiers.
